# A Rare Case of Parotid Nodular Fasciitis in a Six-Month-Old Female

**DOI:** 10.7759/cureus.39459

**Published:** 2023-05-24

**Authors:** Mazin Merdad, Linah Qasim, Mohammed Nujoom, Hani Z Marzouki, Abdulaziz Neazy

**Affiliations:** 1 Department of Otolaryngology-Head and Neck Surgery, King Abdulaziz University, Jeddah, SAU

**Keywords:** fine-needle aspiration, immunohistochemical, self-limiting, proliferative lesion, nodular fasciitis

## Abstract

Nodular fasciitis (NF) is a rare benign self-limiting lesion that is often mistaken for malignancy due to its progressive nature. Reported cases of nodular fasciitis in the parotid gland are uncommon, and its incidence is variable among different age groups. Histopathological and immunohistochemical studies are helpful in distinguishing these kinds of lesions. We report a case of a six-month-old baby with a two-month history of progressive rapid-growing mass in the left parotid region. Clinical examination showed some mild facial nerve weakness with no other significant findings locally or systemically. Fine-needle aspiration (FNA) was inconclusive, and surgical excision was the choice of treatment. On histological examination, the mass was confirmed to be nodular fasciitis, and on follow-up, the patient had no signs of recurrence. Nodular fasciitis can appear in young infants and, if confirmed histopathologically and immunohistochemically, should be treated conservatively.

## Introduction

Nodular fasciitis (NF) is a benign self-limiting pseudosarcomatous myofibroblastic proliferative lesion that affects the muscles, subcutaneous tissue, and fascia [1]. It was first described as a soft tissue tumor by Konwaler et al. [2] in 1955 and was defined as nodular fasciitis in 1966 by Mehregan [3]. Clinically, NF can be easily palpable, but due to its rapidly growing nature, it is often misdiagnosed as a malignant tumor [4]. NF etiology is mostly idiopathic with 5%-10% associated with previous trauma or local injury [3]. They usually affect both genders equally and occur mostly in young and middle-aged individuals [5]. Radiologically, it has unspecific characteristics, making it difficult to confirm the diagnosis [5]. Meanwhile, fine-needle aspiration cytology (FNAC) is often inconclusive, but reports have suggested that the cytomorphology of NF is fairly characteristic, making it possible to diagnose [6]. Moreover, it can have similar cytological traits to that of other benign or malignant tumors, as well as pleomorphic adenoma [7]. In this case, we report a parotid nodular fasciitis finding in a six-month-old patient.

## Case presentation

**Figure 1:**
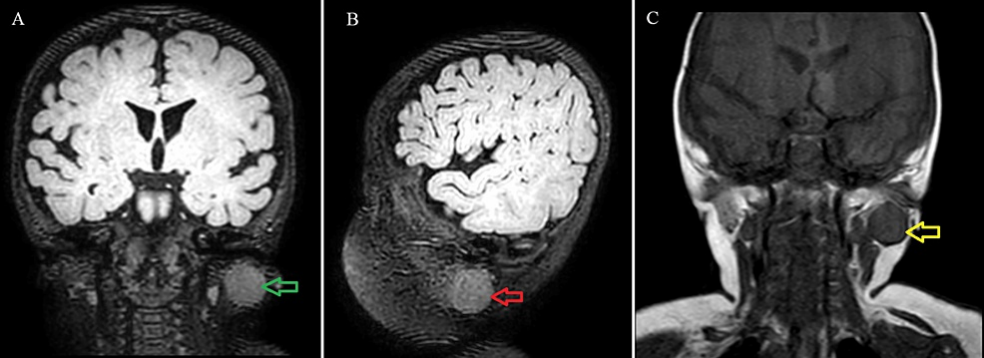
(A) Coronal MRI cut showing a hyper-intense well-circumscribed mass on T1 (green arrow). (B) Sagittal MRI cut showing a hyper-intense well-circumscribed mass on T1 (red arrow). (C) Coronal MRI cut showing a hyper-intense well-circumscribed mass on T2 (yellow arrow). MRI: magnetic resonance imaging

A six-month-old baby female, medically free, presented with a two-month history of left cheek swelling and a preauricular mass, which gradually increased over time. There was no history of trauma, insect bite, local injury, birth defects, or any other systemic complaints. Physical examination revealed a 2.5 cm round, firm, well-circumscribed, and slightly mobile mass, which was palpable on the left cheek. Facial nerve examination revealed a slight weakness that was noticeable on close inspection at the left side with a score of 2 on the modified House-Brackmann scale for infants. Other systemic examinations were otherwise unremarkable. Afterward, laboratory examination data was within normal limits. Ultrasonography (USG) revealed a well-defined, hypoechoic solid mass measuring 2.5 cm × 2 cm with slightly mobile and well-circumscribed borders. Magnetic resonance imaging (MRI) with contrast was performed and showed a well-defined lobulated homogeneous soft tissue mass arising within the left parotid gland measuring 2.5 cm × 2 cm. It demonstrated a bright T2 signal intensity and low T1 signal intensity with mild restricted diffusion (Figure 1).

**Figure 2:**
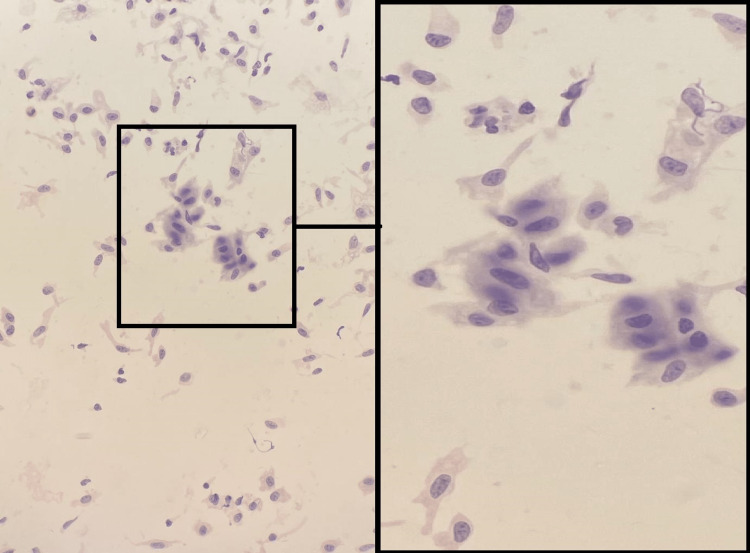
Spindle cells (myoepithelial-like cells) with cytoplasmic cells (epithelial-like cells) with Diff-Quik stain (magnification of 250 and 400).

FNAC was performed and showed a benign neoplasm consistent with pleomorphic adenoma due to the presence of spindle cells (myoepithelial-like cells) with cytoplasmic cells (epithelial-like cells) (Figure 2).

**Figure 3:**
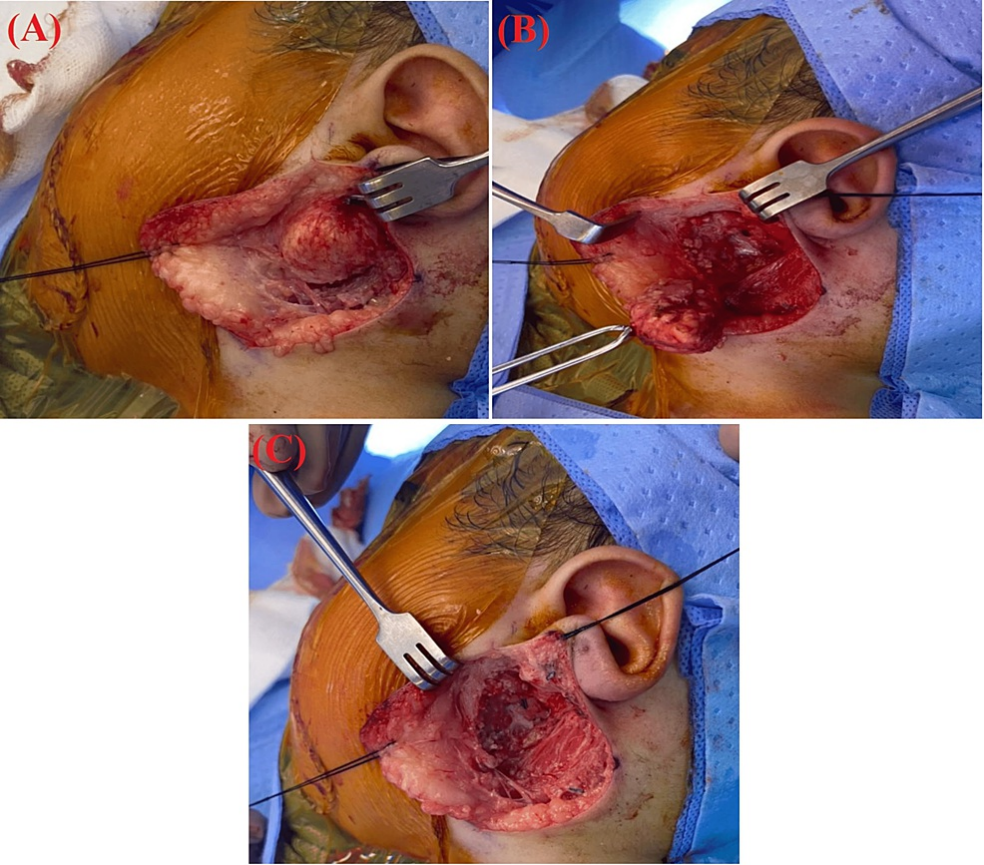
(A) A well-circumscribed lesion is seen superficial to the parotid gland overlying the ramus of the mandible. (B and C) The complete excision of the mass is shown with negative gross margins. (C) Pes anserinus under the mass.

Based on the histopathological finding, a decision to excise the mass was made. As a result, an extracapsular excision was performed with further histological confirmation. On first inspection intraoperatively, the mass was firm and well-defined but was lying on the pes anserinus of the facial nerve and was dissected carefully during the operation (Figure 3).

The nerve was left completely intact, and facial muscles were all stimulated using the nerve integrity monitor. However, the patient did not show any significant facial nerve weakness postoperatively. The histological examination of the excised lesion revealed a proliferation of spindle-shaped cells with myofibroblastic differentiation within a collagenous stroma. The cellular composition demonstrated a lack of significant nuclear pleomorphism and minimal mitotic activity. Additionally, scattered lymphocytes and plasma cells were present within the lesion, indicating an inflammatory component. These findings were consistent with a diagnosis of NF. Meanwhile, the immunohistochemistry examination of the tumor cells was positive for smooth muscle antibody (SMA), indicative of myofibroblastic differentiation and, thus, supportive of the diagnosis. It was negative for anaplastic lymphoma kinase (ALK), β-catenin, epithelial membrane antigen (EMA), S100, desmin, and myogenin. Therefore, no further workup was needed for the patient, just regular follow-ups in the outpatient clinic.

## Discussion

NF is a benign lesion of unknown etiology with some incidents associated with trauma. It is usually self-limiting and is susceptible to multiple recurrences [8]. However, it is very uncommon for NF to occur in parotid glands. Gibson et al. studied 12 patients with NF of the parotid gland and reported that the youngest age was 11 years while the oldest was 70 [8]. They also reported that the majority of the lesions were either superficial to the gland or adjacent peripherally to it. The reported symptoms can also be short from one week or longer reaching up to five months of progressiveness with a mean of 1.9 months [8]. Similarly, our patient presented to the clinic with a two-month duration of progressive and fast-growing lesion.

Cytologically, Gibson et al. reported a mixed finding of fine-needle aspiration (FNA) reports; that is, the most common findings seen are binucleated or multinucleated cells, mitosis, and normal glandular or acinar elements but are separate from the proliferation and negative blending of the epithelium with the spindle cell population [8]. Meanwhile, microscopic features include dense keloid-like collagen, which was reported in seven cases and occasional multinucleated giant cells were identified [8]. On the other hand, histopathological analysis of one reported case of NF found at the canthus showed cellular proliferation of myofibroblastic spindle cells with a tissue culture-like growth pattern [1], while another case of NF at the elbow revealed spindle-shaped polymorphonuclear cells with eosinophilic cytoplasm and vacuoles [9]. These variable reports show that NF is inconsistent and sometimes cannot be distinguishable by FNA and microscopic examination alone. With regard to the use of immunohistochemical analysis, although it was not considered necessary for the diagnosis of nodular folliculitis, its results supported the ruling out of differential diagnoses such as pleomorphic adenoma and smooth muscle tumors as the additional information by evaluating the expression of specific markers in the tissue sample. Similar to our case, the lesional myofibroblastic cells were found to be reactive for SMA and nonreactive for desmin, S100 protein, glial fibrillary acidic protein (GFAP), cluster of differentiation (CD) 117, p63, pan-cytokeratin, CD34, and factor XIIIa [8,9]. Although these immunohistochemical findings are similar to the findings of our case, it is important to note that these additional findings are not specific to NF. Therefore, as in most cases, the definitive diagnosis was primarily made based on the integration of clinical, radiological, and histopathological findings, which serve as the gold standard for NF diagnosis.

The treatment of NF can be conservative because it is a self-limiting lesion. However, due to its fast-growing nature, it is often misdiagnosed as a malignant tumor and is often excised surgically [1,6,7,9]. The recurrence rates of NF in the parotid have been reported to be around 6.7% after excision, but data is still limited due to the rarity of NF in the parotid gland [8]. Surgically, a conservative surgical approach is clinically indicated with limited excision avoiding total parotidectomy. Lastly, the majority of the reported cases that underwent surgical excision in the literature had no recurrence even after five years of follow-up [5,8,9].

## Conclusions

Nodular fasciitis is difficult to diagnose clinically and cytologically. It can occur in any gender, age group, and region. Confusing it with malignant lesions is possible due to its fast progressive growing nature. The histopathological and immunohistochemical examination of the lesion can help guide the diagnosis in the right direction but is not specific. Even though the lesion is self-limiting, limited surgical excision of the lesion is possible with high rates of eliminating any recurrence.
